# Stretchable Sponge Electrodes for Long-Term and Motion-Artifact-Tolerant Recording of High-Quality Electrophysiologic Signals

**DOI:** 10.1021/acsnano.2c04962

**Published:** 2022-07-21

**Authors:** Li-Wei Lo, Junyi Zhao, Kenji Aono, Weilun Li, Zichao Wen, Stephanie Pizzella, Yong Wang, Shantanu Chakrabartty, Chuan Wang

**Affiliations:** † Department of Electrical & Systems Engineering, Washington University in St. Louis, St. Louis, Missouri 63130, United States; § Institute of Materials Science and Engineering, Washington University in St. Louis, St. Louis, Missouri 63130, United States; ‡ Department of Obstetrics & Gynecology, Washington University in St. Louis, St. Louis, Missouri 63130, United States

**Keywords:** stretchable electronics, porous elastomer, porous electrode, electrocardiography, electromyography, uterine contraction monitoring

## Abstract

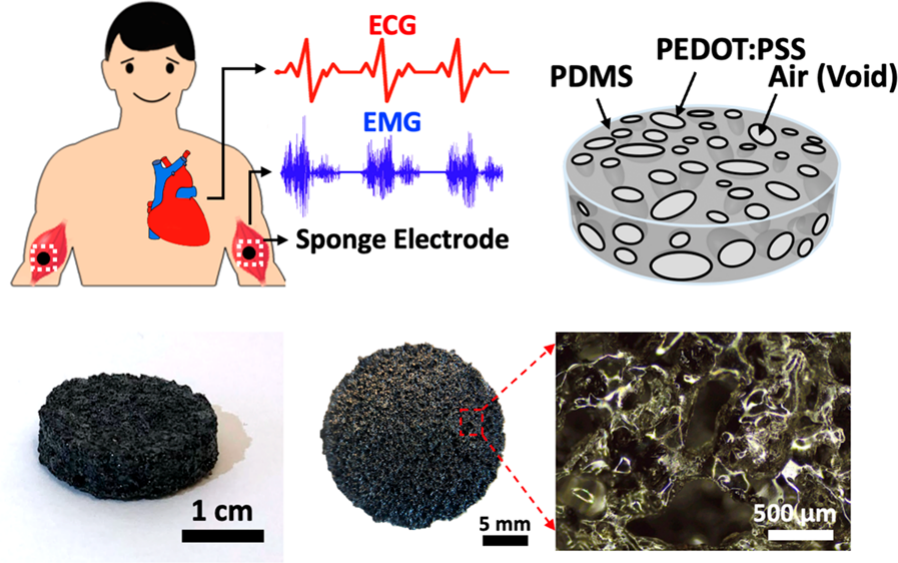

Soft electronic devices and sensors have shown great potential for wearable and ambulatory electrophysiologic signal monitoring applications due to their light weight, ability to conform to human skin, and improved wearing comfort, and they may replace the conventional rigid electrodes and bulky recording devices widely used nowadays in clinical settings. Herein, we report an elastomeric sponge electrode that offers greatly reduced electrode–skin contact impedance, an improved signal-to-noise ratio (SNR), and is ideally suited for long-term and motion-artifact-tolerant recording of high-quality biopotential signals. The sponge electrode utilizes a porous polydimethylsiloxane sponge made from a sacrificial template of sugar cubes, and it is subsequently coated with a poly(3,4-ethylenedioxythiophene) polystyrenesulfonate (PEDOT:PSS) conductive polymer using a simple dip-coating process. The sponge electrode contains numerous micropores that greatly increase the skin–electrode contact area and help lower the contact impedance by a factor of 5.25 or 6.7 compared to planar PEDOT:PSS electrodes or gold-standard Ag/AgCl electrodes, respectively. The lowering of contact impedance resulted in high-quality electrocardiogram (ECG) and electromyogram (EMG) recordings with improved SNR. Furthermore, the porous structure also allows the sponge electrode to hold significantly more conductive gel compared to conventional planar electrodes, thereby allowing them to be used for long recording sessions with minimal signal degradation. The conductive gel absorbed into the micropores also serves as a buffer layer to help mitigate motion artifacts, which is crucial for recording on ambulatory patients. Lastly, to demonstrate its feasibility and potential for clinical usage, we have shown that the sponge electrode can be used to monitor uterine contraction activities from a patient in labor. With its low-cost fabrication, softness, and ability to record high SNR biopotential signals, the sponge electrode is a promising platform for long-term wearable health monitoring applications.

## Introduction

Soft electronic devices and sensors^1−7^ built using elastic materials or structures can maintain electrical performance and reliability under tension or bending conditions, and they have found a wide range of applications in wearable health monitoring devices,^8−10^ resorbable medical implants,^11,12^ human–machine interfaces,^13,14^ and many more. Among the various applications above, clinical or ambulatory monitoring of electrophysiologic signals is widely studied because the biopotential signals transmitted through human skin provide abundant information that can be used for early detection, prevention, and diagnosis of various cardiovascular, muscle, and brain diseases.

For example, electrocardiogram (ECG) is a common diagnostic signal for abnormal cardiac rhythms and electrolyte imbalances which can help prevent heart attacks, strokes, and heart disorders.^15,16^ Electromyogram (EMG) assesses the health of nerves and muscles to diagnose nerve dysfunction or problems with nerve-to-muscle signaling.^17,18^ Electroencephalogram (EEG) monitors brain activity to assess sleep disturbances, brain tumors, and epilepsy.^19,20^ Moreover, EMG or EEG signals collected by stretchable electrodes have also been used to control the wheelchair for patients with spinal cord injuries^21,22^ and prosthetic limb systems for paralyzed patients.^23^

For clinical electrophysiologic signal recording, Ag/AgCl electrodes are still the most commonly used electrodes. Such an electrode consists of a central Ag/AgCl disk and a conductive hydrogel layer that hydrates the nonconductive stratum corneum layer and reduces electrode–skin contact impedance. The commercial Ag/AgCl electrodes do have a few significant drawbacks. First, because the Ag/AgCl electrodes are rigid, they do not conform well to human skin and may result in significant motion artifacts when the patient is active. Second, the Ag/AgCl electrode usually has a small active area and is surrounded by a large area of adhesive or packaging materials that do not contribute to signal collection. Lastly, the conductive gel usually dries within a relatively short period of time (e.g., 1 h) on the electrode surface, and the signal quality will gradually degrade as the gel evaporates. These issues make the commercial Ag/AgCl electrode unsuitable for wearable and long-term ambulatory monitoring applications.

To overcome the above challenges, significant effort has been made to develop lightweight skin-conformable soft electrodes for biopotential recording. For example, some soft epidermal electrodes reported in the literature are designed to reduce the electrode–skin contact impedance, thereby improving signal quality, while others have designed electrodes that stick to the skin, thereby reducing motion artifacts during body movement.^24−28^ One approach is to use microneedles that penetrate the nonconductive stratum corneum layer to reduce the electrode–skin contact impedance. For example, Miura–Ori tessellation structured microneedle electrodes fabricated from soft polydimethylsiloxane (PDMS) substrate coated with a titanium–gold metal layer can bend and maintain stable contact with the skin after penetration.^29^ 3D-printed microneedle electrodes using medical grade 316L stainless steel can bypass the high impedance stratum corneum and increase the overall electrode surface area.^30^ These microneedle electrodes provide stable attachment to the skin and can help obtain reliable ECG and EMG signals. Despite the advantages, there are some safety concerns and debates regarding the use of microneedles due to their invasive nature, which could raise biosafety concerns if the microneedles break into the skin and leave behind materials that could cause redness and skin irritation.^31^ Another approach is to pattern the electrodes into biomimetic microstructures to increase the adhesion between the electrodes and the skin, thereby reducing motion artifacts. Grasshopper-inspired microstructured electrodes made of silver microparticles (AgNPs) mixed with PDMS fabricated on a microstructured wafer have low skin-contact impedance and can be applied directly to the skin without skin preparation or external pressure.^32^ Inspired by the suction mechanism of octopus suckers, octopus-like polymer masters patterned polyurethane (PU)/multiwalled nanotubes (MWNTs)/silver flakes composite can increase the adhesion between electrodes and skin, and these types of electrodes can make conformal contact even with rough and moist human skin and can withstand bending and twisting conditions.^33^ However, these bioinspired approaches typically require a more sophisticated fabrication process to make the mold masters through photolithography and etching processes. A third approach is to modify the surface properties of the electrode to reduce the electrode–skin contact impedance. For example, the modified gold nanoparticle (AuNP) thin film on a polyimide (PI) sheet has been shown to increase the surface roughness, resulting in a 1.54-fold increase in surface area compared to the bare sample.^34^

In this work, we demonstrate a soft sponge electrode that can be fabricated in a low-cost and scalable manner and is ideally suited for long-term and motion-artifact-tolerant recording of high-quality biopotential signals. The sponge electrode has a simple structure comprising a porous PDMS sponge that is thoroughly coated with a conductive poly(3,4-ethylenedioxythiophene) polystyrenesulfonate (PEDOT:PSS) layer. With numerous micropores of hundreds of micrometers inside the sponge, the effective contact area between the skin and the electrode is greatly increased, which leads to a significant drop in skin–electrode contact impedance and an increase in signal-to-noise ratio (SNR). Moreover, compared to conventional planar electrodes, the porous structure of the sponge electrode allows more conductive hydrogel to be stored within the micropores and thus slow the gel drying time and prevent signal degradation over time, making such electrodes suitable for long recording sessions. The gel within the micropores also serves as a buffer layer to help reduce motion between the electrode and the skin and mitigate motion artifacts. Using such sponge electrodes, we have demonstrated high-quality and robust recording of ECG signals and EMG signals from both skeletal muscle cells (biceps contractions) and smooth muscle cells (uterine contractions). This work shows that our sponge electrodes serve as a viable alternative to commercial Ag/AgCl electrodes or other flexible/stretchable thin-film electrodes for wearable ambulatory biopotential monitoring applications.

## Results and Discussion

### Soft Sponge Electrode Fabrication

**Figure 1:**
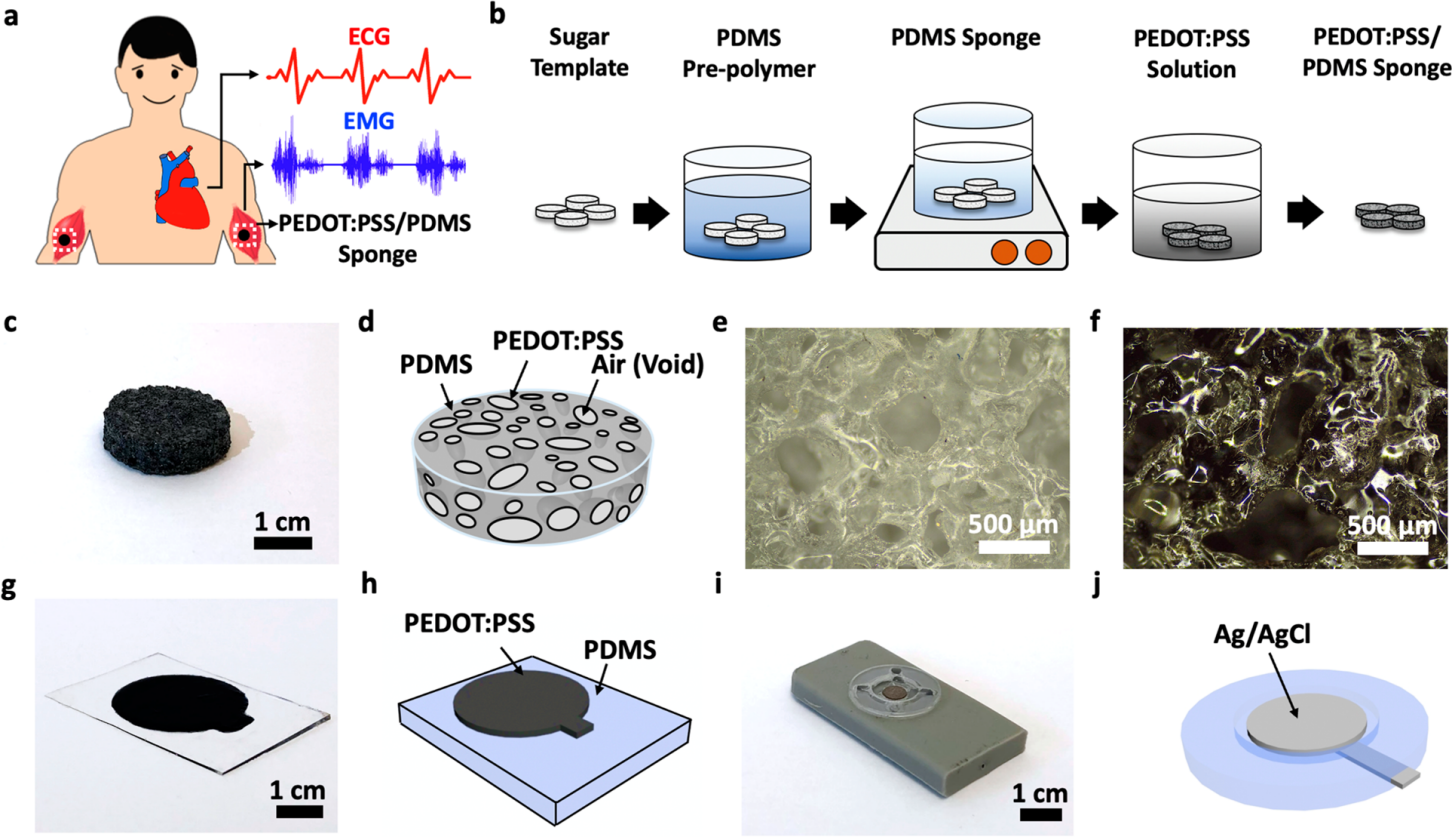
Concept of the soft PEDOT:PSS/PDMS sponge electrode. (a) Schematics illustrating the use of the PEDOT:PSS/PDMS sponge electrode for ECG and EMG recording applications. (b) Schematic diagrams illustrating the fabrication steps used to make the sponge electrodes. (c) Photograph of a sponge electrode. (d) Schematic illustrating the structure of the sponge electrode. (e, f) Optical micrographs showing the microstructures of the PDMS sponge (e) before and (f) after the PEDOT:PSS coating. (g, h) Photograph and schematic diagram of the PEDOT:PSS thin-film directly printed on a piece of planar PDMS substrate. (i, j) Photograph and schematic diagram illustrating the structure of a commercial Ag/AgCl electrode used as a gold-standard reference in this study.

As illustrated in Figure 1a, the porous PEDOT:PSS/PDMS sponge can be used as a soft wearable electrode to record high-quality bioelectrical signals owing to its large internal surface area. For example, when two electrodes are placed on the right and left arms, ECG signals showing the electrical activities during the cardiac cycle can be obtained. When two electrodes are attached to the biceps, the EMG signals generated from the electrical activities that occur in response to nerve stimulation of the muscle can be recorded. The fabrication process of the porous PEDOT:PSS/PDMS electrode is schematically illustrated in Figure 1b, which begins with molding commercially available white sugar cubes into sacrificial templates with the desired sizes and shapes. The sugar templates are then immersed into the liquid PDMS, which then solidifies after curing. The sugar/PDMS templates are subsequently placed in a hot water bath to allow the sugar particles to be dissolved, leaving behind the porous PDMS templates. To facilitate the wetting of the PEDOT:PSS solution onto the hydrophobic PDMS surfaces, the porous PDMS templates need to be pretreated with oxygen plasma. After treatment, the porous PDMS templates are immersed into the PEDOT:PSS solution followed by annealing of the PEDOT:PSS thin film. After the steps above, the porous PDMS templates are thoroughly coated with a conductive PEDOT:PSS thin film to form the soft sponge electrode. More details about the fabrication processes can be found in the Methods. The photo and schematic diagram of the sponge electrode are shown in Figure 1c,d, and its softness is illustrated in Figure S1. As shown in the optical micrographs of the PDMS template before (Figure 1e) and after (Figure 1f) the PEDOT:PSS dip-coating process, the color of the PDMS sponge turns from white to black, confirming that the numerous micropores inside the PDMS template are coated with a conductive PEDOT:PSS thin film, allowing the effective surface area of the electrode to be significantly increased. The size of these micropores ranges from 300 to 500 μm, which can be directly controlled by the size of the sugar particle used.

### Electrode-Skin Impedance Analysis

In order to thoroughly understand the benefits offered by a porous electrode for wearable and clinical electrophysiologic signal recording applications, we have systematically compared the sponge electrode against the screen-printed planar PEDOT:PSS electrode (Figure 1g,h) and the gold-standard commercially available Ag/AgCl electrode (Figure 1i,j). The detailed analysis and comparison of electrode–skin impedance, ECG and EMG signal quality, motion artifacts, and signal decay during long-term recording will be discussed in the following sections.

**Figure 2:**
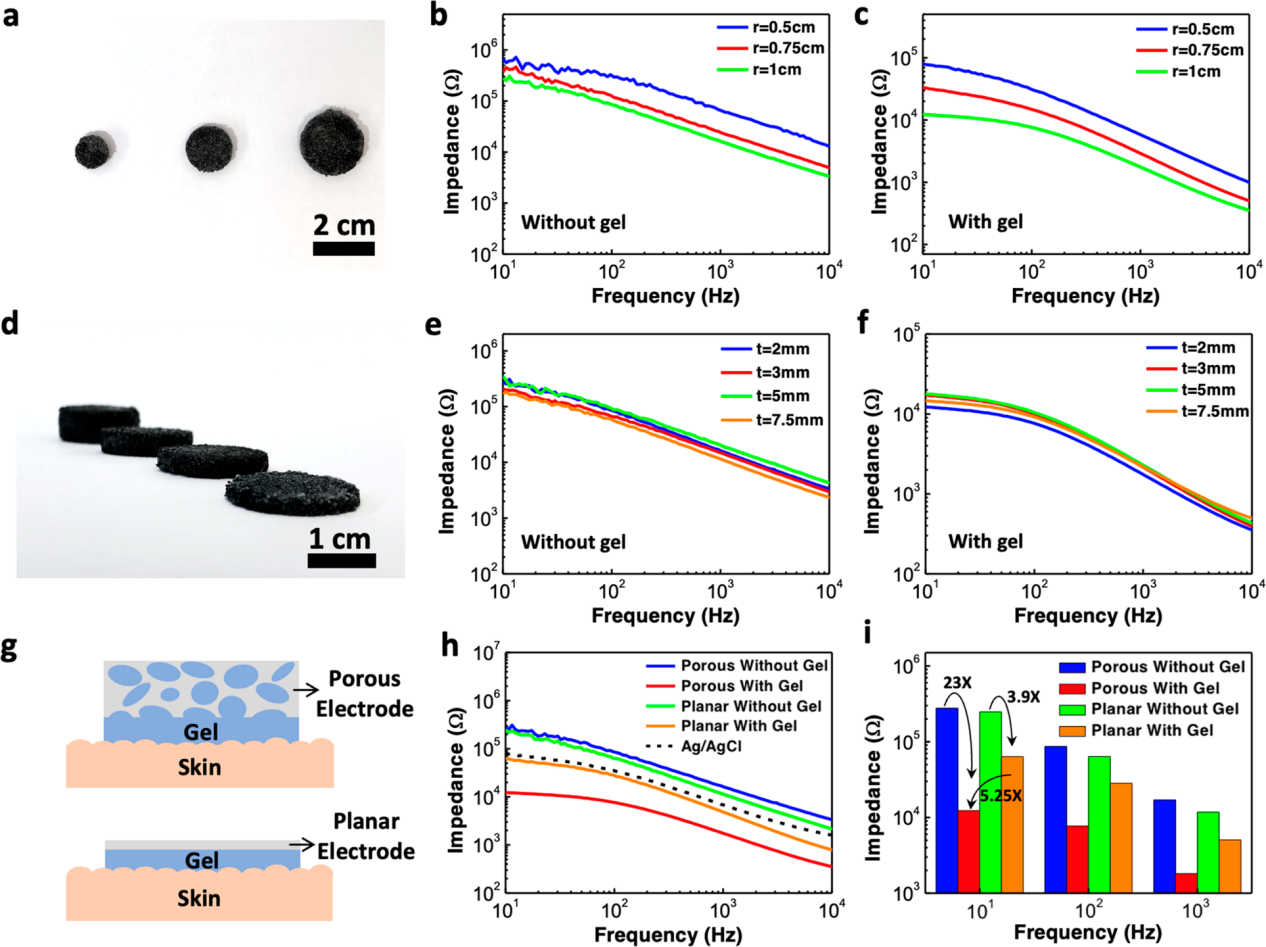
Skin-electrode impedance characterization of the porous PEDOT:PSS/PDMS electrode. (a) Photograph showing three sponge electrodes with the same thickness of 2 mm and different radii of 0.5, 0.75, and 1 cm. (b, c) Impedance spectra measured using the sponge electrodes of different radii (b) without and (c) with the use of conductive hydrogel. (d) Photograph showing four sponge electrodes with the same radius of 1 cm and different thicknesses of 2, 3, 5, and 7.5 mm. (e, f) Impedance spectra measured using the sponge electrodes of different thicknesses (e) without and (f) with the use of conductive hydrogel. (g) Schematic diagrams illustrating the difference in the electrode-gel-skin contact area between the sponge electrode and planar electrode. (h) Comparison of impedance spectra measured using the sponge electrode, planar electrode, and commercial Ag/AgCl electrode. (i) Impedance values at 10, 100, and 1000 Hz for the porous electrode and planar electrode with and without the use of conductive hydrogel.

The electrode–skin contact impedance of the sponge electrode was measured by placing two circular-shaped electrodes on the skin surface of the human subject’s arm with a separation distance of 5 cm (Figure S2). We first investigated the effect of the sponge electrode size and thickness on the electrode–skin impedance. As shown in Figure 2a, sponge electrodes with a fixed thickness of 2 mm and a radius ranging from 0.5 to 1 cm were fabricated. Parts b and c of Figure 2 are their measured electrode–skin impedance from 10 Hz to 10 kHz without and with the use of conductive hydrogel, respectively. Similarly, sponge electrodes with a fixed radius of 1 cm and different thicknesses ranging from 2 to 7.5 mm were also studied (Figure 2d), and their corresponding electrode–skin impedance curves are presented in Figure 2e,f. Table 1 summarizes the key results and shows how the impedance scales with electrode area and thickness. As the radius/area of the sponge electrode increases, the impedance decreases monotonically, both with and without gel, which is as expected. However, the scaling is not exactly inversely proportional to the area and it can be attributed to the micropores inside the sponge. For example, as the radius changes from 0.5 to 1 cm, the area of the electrode increases by a factor of 4 but the impedance without gel only decreases from 678.6 to 277.5 kΩ or a factor of 2.45. This is because when there is no conductive gel applied, only the top surface of the electrode contributes to the electrode–skin interface conductance, and due to those pores on the surface, not the entire top surface is in contact with the skin, which causes the scaling behavior to deviate slightly from the ideal case. After the sponge electrode was filled with conductive hydrogel, the electrode–skin impedance dropped significantly to as low as 12.2 kΩ at 10 Hz for the sample with a 1 cm radius, corresponding to a 22.7-fold reduction in impedance after gel. The decrease in electrode–skin contact impedance can be attributed to two factors: (1) the drastic increase in effective electrode–skin contact area due to the internal surfaces of the micropores inside the sponge electrode and (2) the penetration and hydration of the conductive hydrogel into the stratum corneum layer, the outermost layer of the epidermis consists of dead skin cells, which are relatively nonconductive due to the lack of water in the cells.^35^

**Table 1:** Effect of the Electrode Area and Thickness on the Electrode-Skin Impedance

Area Scaling				Thickness Scaling				
Radius (cm)	0.5	0.75	1	Thickness (mm)	2	3	5	7.5
Area (cm^2^)	0.79	1.77	3.14					
Impedance without gel @ 10 Hz (kΩ)	678.6	366.8	277.5		277.5	205.7	348.1	168.5
Impedance with gel @ 10 Hz (kΩ)	80.2	33.3	12.2		12.2	17.2	17.7	14.6

Regarding the thickness of the electrode, ideally, the impedance should increase linearly with thickness for a solid piece of conductor. On the other hand, the case for a porous conductor is much more complicated as an increase in thickness also leads to a further increase in the effective contact area between the skin and the PEDOT:PSS, which in turns helps lower the impedance. From the results in Table 1, it was observed that the measured impedance is largely independent of the electrode thickness, especially after the conductive gel was applied. The results suggest that while the entire sponge is filled with conductive gel, it is likely that the electrical conduction only happens down to a certain depth, and thus, further increase of electrode thickness beyond this depth would not result in more performance gain. We have established a model to estimate the porosity, inner surface area, and expected reduction in sponge electrode impedance and those detailed analysis can be found in Supporting Information Notes S1–S3. To sum up, the electrode–skin contact impedance is area-dependent but thickness-independent. For the following experiments on ECG and EMG signal recording, we selected the sponge electrode with a radius of 1 cm and a thickness of 2 mm.

In order to further illustrate the benefit offered by the porous sponge electrode, we compare it with a screen-printed PEDOT:PSS thin-film electrode on a planar PDMS substrate as well as a gold-standard BioSemi Ag/AgCl electrode widely used in clinical recording sessions. Figure 2g schematically illustrates the advantages offered by a porous electrode compared to conventional planar electrodes. Because the conductive gel soaks up the entire sponge electrode and makes electrical contact with the PEDOT:PSS that is coated on the inner surfaces of the micropores, the skin–electrode contact area is significantly larger compared to a planar electrode, resulting in a decrease in electrode–skin contact impedance. The impedance measurement results in Figure 2h confirm the above. Before the conductive gel was applied, the measured electrode–skin impedance from the porous PEDOT:PSS electrode and planar PEDOT:PSS electrodes of the same size were 277 kΩ (blue trace) and 247 kΩ (green trace) at 10 Hz, respectively. The impedance from the planar electrode was actually slightly lower than that of the sponge electrode, which could be attributed to the fact that the bottom surface area of the sponge electrode was only partially in contact with the skin due to the pores resulting in a higher impedance (Figure S3). Once the conductive hydrogel was applied, the impedance of both the porous and the planar electrode decreased significantly by a factor of 23 to 12 kΩ (red trace) and a factor of 3.9 to 63 kΩ (orange trace), respectively. Compared to the planar electrodes, the use of porous electrodes helps reduce the skin–electrode impedance by a factor of 5.25 at a frequency of 10 Hz. Figure 2i summarizes the electrode–skin contact impedance of the porous and planar electrodes at various frequencies, which shows that the drop in impedance after the application of the conductive hydrogel is much more significant in the sponge electrode (blue to red) compared to the planar electrode (green to orange) for all frequencies tested. More importantly, the porous electrode also offers a much lower impedance compared to the commercial Ag/AgCl electrode (80.4 kΩ at 10 Hz according to the dashed line in Figure 2h), suggesting the great potential of using the porous electrode for wearable or clinical electrophysiologic signal recording applications. The low skin–electrode interface impedance could lead to improved SNR as will be discussed in the following sections. Lastly, because the porous electrode is mechanically soft, it can function properly even when stretched. We have measured the skin–electrode impedance of the porous electrode under different tensile strain levels up to 20% and observed negligible change in skin–electrode impedance as shown in Figure S4.

### Sponge Electrode for Motion-Artifact-Tolerant ECG Recordings

**Figure 3:**
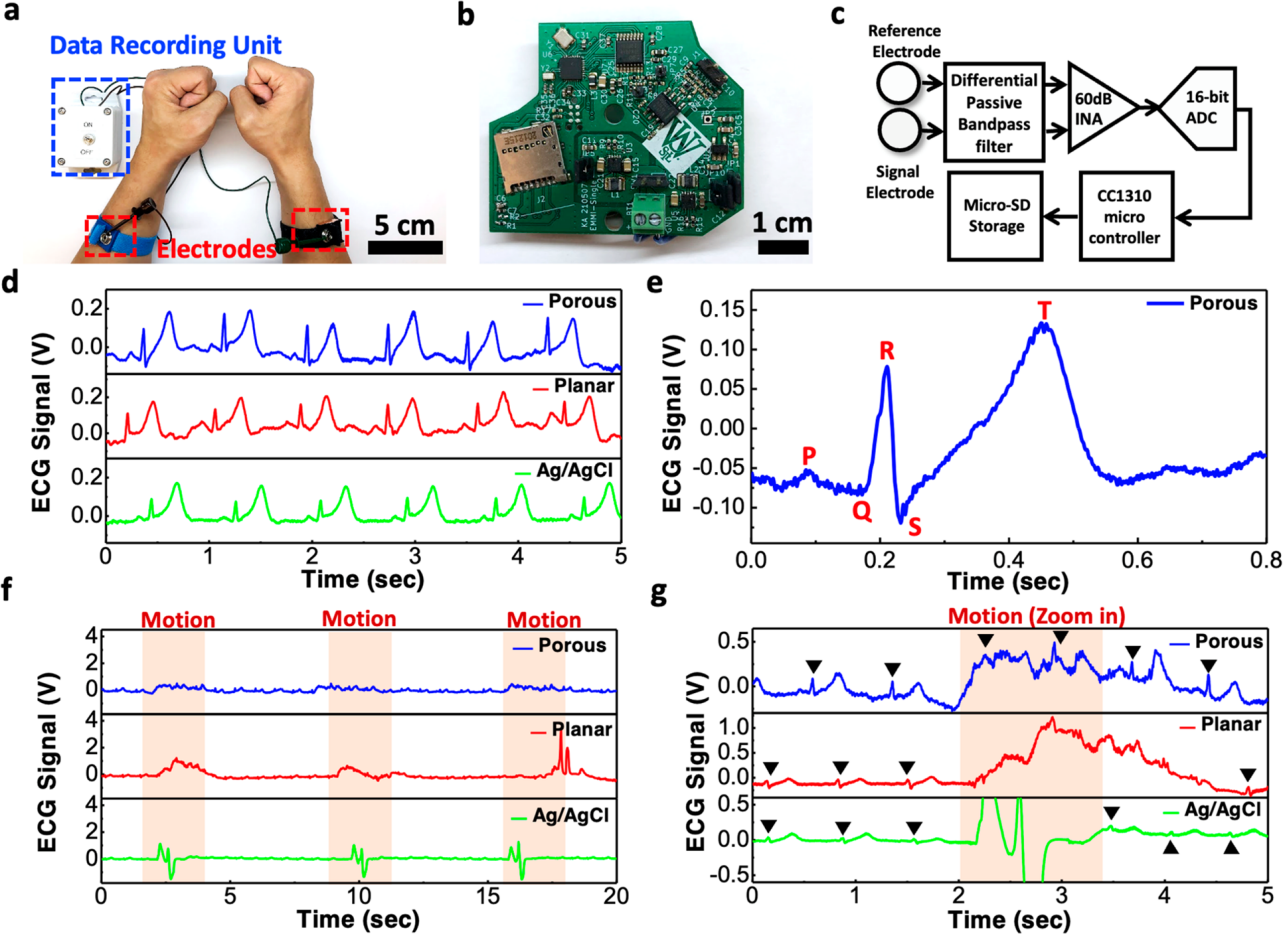
ECG recording and the effect of motion artifacts. (a) Photograph showing the ECG recording setup. (b) Photograph showing the PCB board of our in-house built biopotential data recorder. (c) Block diagram of the portable data recording unit. (d) ECG signals measured using the porous PEDOT:PSS/PDMS electrode, planar PEDOT:PSS electrode, and commercial Ag/AgCl electrode. (e) Representative cycle of the ECG waveform acquired by the sponge electrode showing clear P wave, QRS complex, and T wave. (f) ECG signals measured under the presence of motion artifacts caused by periodic body movement. (g) Zoomed-in view of the data in (f) showing the ECG peaks along with the motion artifacts.

The porous PEDOT:PSS/PDMS electrode can be used for high-quality electrophysiologic signal recording. As shown in Figure 3a, during the ECG experiment, two sponge electrodes filled with conductive hydrogel were used as the signal and reference electrodes and were placed on a volunteer’s left and right arms and fastened using antistatic wrist straps. The electrodes were wire-connected to an in-house built data recording unit with a sampling rate of 1 kHz that can either store the recorded data directly into a memory card for optimal power consumption or wirelessly transmit the signal to a computer in real-time. A photograph of the PCB board inside the data recording unit and its block diagram are shown in Figure 3b,c, respectively. The recorded signals are first filtered through a differential passive bandpass filter with a frequency band of 0.1 Hz to 1 kHz to remove unwanted signals. The filtered signal is then amplified by a 60 dB instrumentation amplifier and digitized using a 16-bit analog-to-digital converter, followed by a microcontroller that handles and stores the digital signal into a microSD card. Using the above setup, we measured the ECG signals on the same volunteer, during the same day, and within a relatively short period of time using the porous and planar PEDOT:PSS electrodes and commercial Ag/AgCl electrodes. The recorded data in Figure 3d show clear ECG signals from all three types of electrodes with periodic peaks representing the different phases of the electrical activities during a heartbeat. The ECG signals recorded by the porous electrodes under stretched conditions can be found in Figure S5. From the period of the ECG waveform, one can estimate the corresponding heart rate to be ∼75 bpm. Figure 3e shows a representative cycle of the ECG waveform measured by the porous electrode, which exhibits a clearly distinguishable P wave that arises from the atrial depolarization, the QRS complex that represents ventricular depolarization, and the T wave that reflects the ventricular repolarization. These waveforms can be further used to detect cardiac abnormalities and various cardiovascular diseases.^36^ It is worth noting that the T wave amplitude is slightly elevated compared to the R-peak, which can be attributed to the high-pass and low-pass filtering stages in the frontend electronics of our in-house built data recording unit, causing certain frequency ranges to be amplified and the shape of the ECG signal to be slightly distorted.

As shown previously in Figure 2g, because the conductive gel penetrates the micropores inside the sponge electrode to form an intimate electrical contact between the PEDOT:PSS conductive layer and the skin surface, the large internal surface area offered by the sponge electrode and the interfacial layer offered by the gel can also help mitigate motion artifacts. Generally speaking, motion artifacts could be caused by either the relative motion of the electrodes with respect to the skin, or triboelectric effects that cause charge accumulation at the interface of the electrodes. For the sponge electrode, its soft and porous structure could serve as a shock absorber to reduce motions between the electrode and the skin, and the conductive gel absorbed into the micropores could also serve as a buffer layer to help keep the contact area between the skin and the recording electrode fairly constant and equalize any charge that might be formed at the interface. For these reasons, the sponge electrodes are expected to be more robust to motion artifacts as confirmed by the experiments. Figure 3f shows the recorded ECG waveforms when strong motions (human subject rapidly standing up and sitting down) were intentionally introduced during the regions indicated by the orange color. Note that the vertical axis range is from 0 to 4 V, which is significantly greater than the ECG signal amplitude to allow the motion artifacts to be seen. When motions were present, the muscle activities and the movement of the electrode relative to the skin surface resulted in strong voltage spikes exceeding 1 V in the ECG waveforms recorded by both the planar electrodes and the commercial Ag/AgCl electrodes. In contrast, the motion artifacts were significantly mitigated in the waveforms recorded by the porous electrodes, as shown in the blue trace. Figure 3g shows a zoomed-in portion of the data in Figure 3f, in which the periodic R-peaks of each ECG waveform are indicated by the black triangle marks. Comparing the three waveforms, one can see that the ECG signals remained visible under the influence of motion artifacts for the porous electrode but disappeared for the planar and Ag/AgCl electrodes, again suggesting that the porous electrode is capable of motion-artifact-tolerant ECG recording, which is crucial for wearable devices and ambulatory electrophysiology recording sessions.

### Long-Term Monitoring of ECG Signals

**Figure 4:**
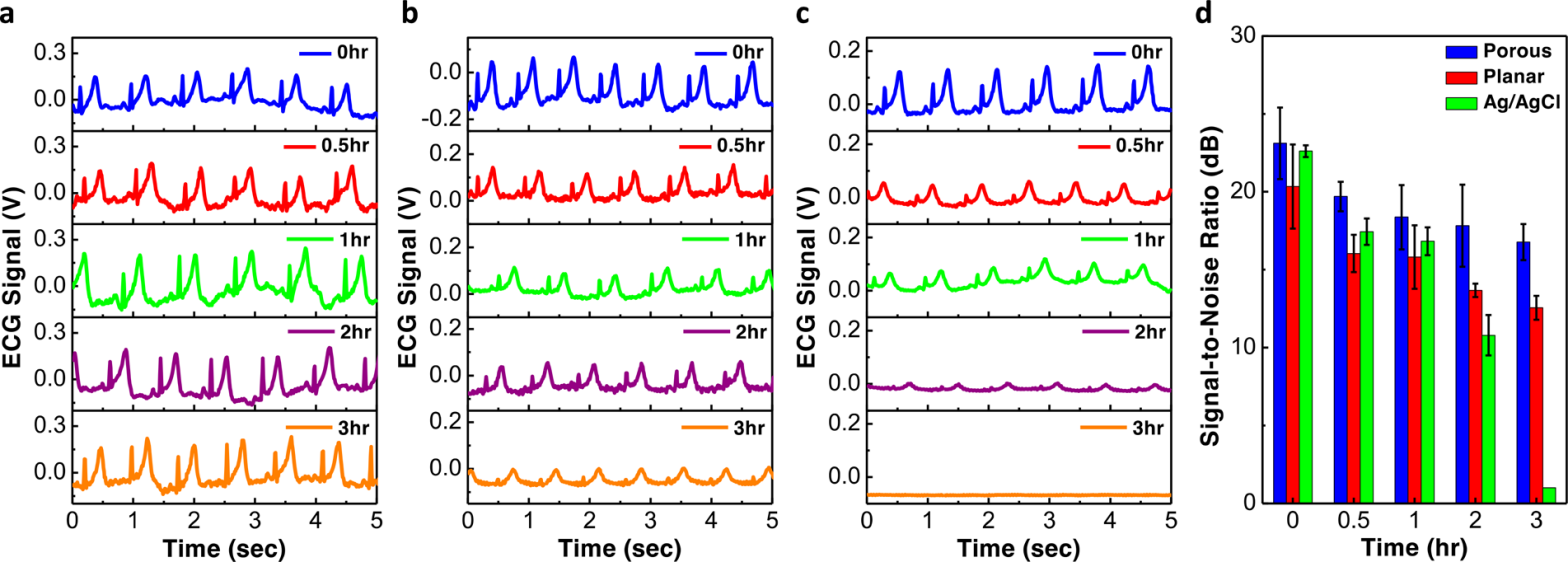
Long-term ECG signal recording. (a–c) ECG signals measured after various amount of time using the (a) porous PEDOT:PSS/PDMS electrodes; (b) planar PEDOT:PSS electrodes; and (c) commercial Ag/AgCl electrodes. (d) Comparison of the SNR between the three different types of electrodes.

For electrophysiologic signal recording sessions in a clinical setting using conventional electrodes, the duration is typically limited to less than 1 h due to the drying of the conductive hydrogel. The signal quality could degrade significantly as the gel dries up. Unlike the conventional electrodes, the sponge electrode could hold much more gel inside the micropores and thus allow the electrodes to last much longer. Figure 4 compares the ECG waveforms measured by the porous, planar, and commercial electrodes over a duration of 3 h. As shown in Figure 4a, even after 3 h, the porous electrodes kept producing high-quality ECG signals with clear P wave, QRS complex, and T wave. As for the planar electrodes (Figure 4b) and commercial Ag/AgCl electrodes (Figure 4c), the signals were good from the beginning but degraded gradually over time. After 3 h, the signal amplitude dropped significantly for the planar electrode or even completely disappeared for the Ag/AgCl electrode. Such signal decay can be attributed to the drying of gel as shown in Supporting Information Figures S6 and S7. Because the total volume of the conductive hydrogel held inside the porous electrode is much larger compared to the amount of gel on the top surface of the planar electrodes, the evaporation of the gel is much slower and thus allows the porous electrode to last longer. We also extracted the SNR of the ECG waveforms, and the results are displayed in Figure 4d. In the beginning, the highest SNR was 23.1 dB from the porous electrodes, which was just slightly better compared to the 22.6 dB from the commercial Ag/AgCl electrodes and 20.3 dB from the planar electrodes. After 2 h of recordings, the SNR of the Ag/AgCl and planar electrodes dropped significantly to 10.7 and 13.6 dB, respectively, while the porous electrode was still able to maintain an SNR of 17.8 dB. After 3 h, the signals from the Ag/AgCl electrodes disappeared, and the SNR from the planar electrode dropped to 11.7 dB, worse than the 16.1 dB offered by the porous electrodes. In conclusion, the results suggest that the evaporation rate of the conductive hydrogel may be slower on the PEDOT:PSS surfaces compared to the Ag/AgCl surfaces and the porous structure inside the sponge electrode can store a larger amount of gel, allowing the stratum corneum layer to remain hydrated and maintaining good electrical contact between the skin and the electrode.

### Recording of Muscle EMG and Uterine Contraction EMG Signals

**Figure 5:**
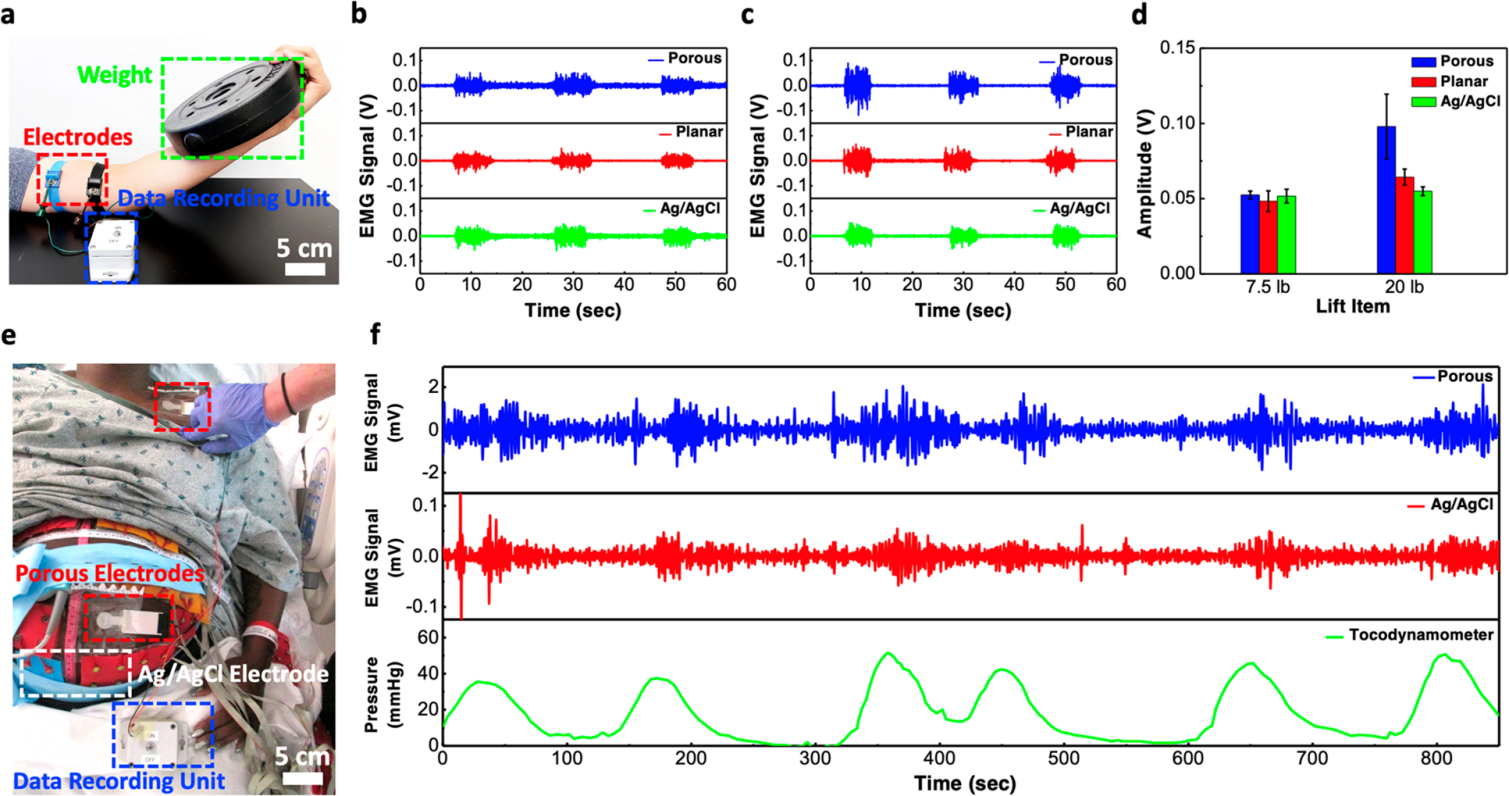
EMG signal recording from skeletal muscle and smooth muscle. (a) Photograph showing the setup for measuring EMG signal from the contraction of biceps. (b, c) EMG signals measured using various kinds of electrodes when the subject was lifting a (b) 7.5-lb or (c) 20-lb weight. (d) Comparison of the EMG signal amplitude measured with sponge, planar, and commercial Ag/AgCl electrodes. (e) Photograph showing the setup for recording EMG signals from uterine contraction activities in a clinical setting. (f) Comparison of EMG waveforms recorded from our porous electrodes and in-house built data recorder, the commercial BioSemi active Ag/AgCl electrodes and BioSemi biopotential measurement system and the corresponding uterine contractions recorded from a tocodynamometer.

The porous electrode can also be used to measures electrical activities in response to a nerve stimulation of the muscle fibers. In Figure 5a, two porous electrodes separated by a distance of 5 cm were placed on the biceps of the left arm and the same data recording unit above was used for data collection. When the volunteers lifted a weight plate, the biceps brachii muscle contracted and produced strong EMG signals. Figure 5b shows the EMG waveforms recorded by the porous, planar, and Ag/AgCl electrodes when the volunteer lifted a 7.5 lb weight. The amplitudes of the EMG signal measured by the porous, planar, and Ag/AgCl electrodes were 0.052, 0.048, and 0.051 V, respectively. When the weight was increased to 20 lbs, the amplitude of the EMG signal measured by the porous, planar, and Ag/AgCl electrodes increased to 0.098, 0.064, and 0.054, respectively (Figure 5c). From the EMG signal amplitude summarized in Figure 5d, one can find that the porous electrodes can record similar or better signals compared to the gold standard Ag/AgCl electrodes, especially when lifting heavier weight that induces stronger muscle contraction. From the previous experiments on ECG motion artifacts, we have shown that the porous electrode can capture the ECG signal even during the presence of significant motion and muscle activities. Similarly, when heavy weights are lifted, the muscle may shake and it can affect the EMG signal recording. With the use of a sponge electrode, the conductive hydrogel in the micropores could help mitigate the motion artifacts induced by the muscle shaking, thus allowing a more accurate EMG signal to be recorded.

In addition to measuring EMG signals from skeletal muscle cells, the sponge electrodes can also be used to measure EMG from smooth muscle cells that produce much weaker signals. For example, high-quality signals induced by the uterine contraction activities can be measured from the abdominal surface of pregnant women, providing a noninvasive method for evaluating various labor-related risks such as preterm birth.^37−40^Figure 5e and Figure S8 show the placement of the sponge electrodes on a pregnant woman in labor during the clinical recording session. The sponge electrodes were connected to our own data recording unit, and the commercial active Ag/AgCl electrodes were connected to a commercial 280-channel 24-bit resolution biopotential measurement system (BioSemi B.V, Amsterdam, The Netherlands) for data recording. The recording session lasted a total of approximately 850 s or 14 min, with six uterine contractions confirmed by the tocodynamometer (TOCO). Figure 5f shows the EMG data simultaneously recorded by the sponge and Ag/AgCl electrodes as well as the TOCO data that shows the pressure created by the uterine contraction activities. From the figure, one can see that the EMG signals recorded by the sponge electrode closely resembled the ones recorded by the gold-standard commercial BioSemi Ag/AgCl active electrodes, and both EMG waveforms correlated well with the uterine contractions recorded by the TOCO. Moreover, the signals from the sponge electrodes exhibited much larger amplitude and better SNR compared to the Ag/AgCl electrodes, which was in part due to the higher gain from our in-house data recording unit (60 dB) compared to the BioSemi data recording system and also the larger surface area offered by our porous electrode.

## Conclusion

In conclusion, we have developed a simple and low-cost method for fabricating a soft sponge electrode that is composed of a porous PDMS sponge coated with a conductive PEDOT:PSS layer and demonstrated its use for high-quality ECG and EMG signal recording applications. The porous structure greatly increases the skin–electrode contact area, thereby lowering the skin–electrode impedance and resulting in high-quality biopotential signal recording with improved SNR. More importantly, the sponge electrode can hold significantly more conductive hydrogel within its micropores to help mitigate motion artifacts and allow the electrode to be used for long recording sessions, both of which are crucial for wearable and ambulatory monitoring applications. This work demonstrates the great potential of our sponge electrode as a low-cost alternative for recording high-quality electrophysiologic signals in both in-home and clinical usage settings.

## Methods

### Materials

PDMS (Sylgard 184) was purchased from Dow Corning. PEDOT:PSS (1.3 wt % dispersion in H_2_O, conductive grade), PEDOT:PSS (5 wt % conductive screen printable ink), bis(trifluorometh- ane)sulfonimide lithium salt, and ethylene glycol (anhydrous, 99.8%) were purchased from Sigma-Aldrich. White sugar cubes were purchased from C&H.

### Preparation of the Porous PDMS Template

The white sugar cubes 1.5 (W) x 1.5 (L) x 1.5 cm (H) were first placed into various molds to form desired shape and size. The PDMS was prepared by mixing the PDMS prepolymer with the curing agent with a mixing ratio of 10:1 w/w. A vacuum desiccator was used to remove air bubbles from the PDMS liquid. The sugar templates were then dipped into the PDMS liquid for 2 h to let the pores to be fully filled with the PDMS liquid and the sugar templates were subsequently cured in the oven for 3 h at 80 °C. After that, the sugar templates were placed in a hot water bath for 1 h at 100 °C to dissolve the sugar particles and form the porous PDMS template.

### Preparation of the Porous PEDOT:PSS/PDMS Electrode

The porous PDMS template was pretreated by oxygen plasma at 60 W for 30 s for the top and bottom surfaces to aid the wetting of the ink. Then 5 wt % of ethylene glycol was added into PEDOT:PSS solution and then stirred at room temperature for 1 h. After that, the porous PDMS template was then dipped into the as-prepared PEDOT:PSS solution for 30 min. The PEDOT:PSS-coated porous PDMS sponge was then cured in the oven for 3 h at 80 °C.

### Preparation of the Planar PEDOT:PSS Electrode

The PDMS substrate was prepared by mixing the PDMS prepolymer with the curing agent with a mixing ratio of 10:1 w/w. A vacuum desiccator was used to remove air bubbles from the PDMS liquid. Two glass slides with a spacer was used to cast a 0.5 mm thick PDMS substrate. The PDMS substrate was then cured in the oven for 3 h at 80 °C. To aid the wetting of the ink, the PDMS substrate was treated by oxygen plasma at 30 W for 15 s. Then 10 wt % of bis(trifluoromethane)sulfonimide lithium salt was added into PEDOT:PSS solution (5 wt %, conductive screen printable ink) and stirring rigorously for 10 min. After that, the PEDOT:PSS thin film was screen-printed onto the pretreated PDMS substrate by using a 3D-printed shadow mask with opening of 2 cm in diameter and 0.3 mm in thickness. The printed PEDOT:PSS electrodes were then placed on a hot plate for 3 h at 80 °C.

### Characterization of Porous PEDOT:PSS/PDMS Electrode

Optical microscope (Olympus BX53M) was used to capture the microstructure of the porous PDMS and the porous PEDOT:PSS/PDMS. An impedance analyzer (Bode 100, OMICRON Lab) was used to measure the impedance of various types of electrodes by placing two electrodes on a human subject’s arm separated by 5 cm. Unless otherwise specified, the pores within PEDOT:PSS/PDMS electrode were filled with conductive electrolyte gel (Parker Laboratories). All electrode comparison studies were performed on the same person and on the same day.

### Electrophysiologic Signal Measurement

Electrophysiologic signals were measured using the in-house built data recording unit with a sampling rate of 1 kHz that stored the data directly into a memory card. ECG signals were recorded by placing one electrode on the right arm and the other on the left arm. Muscle EMG signals were recorded by placing two electrodes on the biceps muscle of the left arm, separated by 5 cm. Uterine EMG signals were recorded by placing one electrode on the anterior abdominal surface and the other on the shoulder of the pregnant woman.

### Statistical Analysis of the SNR

A Python script was used for signal processing and calculating the SNR. For ECG signals, the signal processing is composed of a 60 Hz notch filter and 200 Hz 10th order Butterworth low-pass filter. The SNR was calculated using the equation SNR (dB) = 20  where *A*_signal_ is the peak-to-peak voltage of the ECG signal and *A*_noise_ is the peak-to-peak voltage of the noise. For muscle EMG signals, the signal processing is composed of a 60 Hz notch filter, a 450 Hz low-pass filter and a 20 Hz high-pass filter. Uterine contraction EMG signals were processed by a 60 Hz notch filter, a 1 Hz low pass filter, and a 0.34 Hz high pass filter.

### Patient Study

The patient study was carried out at the Barns and Jewish Hospital, Washington University School of Medicine in St. Louis, and the Washington University in St. Louis Institutional Review Board approved this study (IRB ID No. 201612140). One multiparous patient was recruited for this study, and the patient signed the informed consent documents. The uterine contraction EMG recording was performed in a labor and delivery room in Barns and Jewish Hospital, when the patient was in active labor with 4.5 cm cervical dilation.

## Supporting Information Available

Details and softness of the sponge electrode; placement of the sponge electrodes for electrode–skin contact impedance measurement; difference in skin–electrode contact area between the porous electrode and planar electrode when no conductive hydrogel is used; skin–electrode impedance characterization of the porous electrode under different tensile strain levels; ECG waveforms recorded by the porous electrodes under stretched condition; porous electrode before and after application of the conductive gel; comparison of gel drying time on porous electrode and planar electrode; clinical recording of the electrophysiologic signal from uterine contraction activities using the sponge electrode; calculation of the internal surface area of all micropores inside a porous electrode; calculation and comparison of the surface area from a single layer micropores in a porous electrode and the surface area of a conventional planar thin-film electrode; analysis of the decrease in skin–electrode impedance and increase in surface area offered by the porous electrode compared to the planar electrode (PDF)
