# Supplementary material for: Stretchable Sponge Electrodes for Long-Term and Motion-Artifact-Tolerant Recording of High-Quality Electrophysiologic Signals
Source: ACS Nano. 2022 Jul 21;16(8):11792–801. doi: 10.1021/acsnano.2c04962 (PMC9413418; doi:10.1021/acsnano.2c04962)
Supplement: Supplementary file 1 — nn2c04962_si_001.pdf [file nn2c04962_si_001.pdf]

Stretchable Sponge Electrodes for Long-Term and Motion-Artifact-Tolerant Recording of High-Quality Electrophysiologic Signals

Li-Wei Lo,[†,§] *Junyi Zhao,*[†] *Kenji Aono,*[†] *Weilun Li,*[†] *Zichao Wen,*[‡] *Stephanie Pizzella,*[‡] *Yong Wang,*[†,‡] *Shantanu Chakrabartty,*[†] *and Chuan Wang*[*,†,§]

[†] Department of Electrical & Systems Engineering, Washington University in St. Louis, St. Louis, Missouri 63130, United States

[§] Institute of Materials Science and Engineering, Washington University in St. Louis, St. Louis, Missouri 63130, United States

[‡] Department of Obstetrics & Gynecology, Washington University in St. Louis, St. Louis, Missouri 63130, United States

[*] Corresponding author: chuanwang@wustl.edu

Supporting Information

Details and softness of the sponge electrode

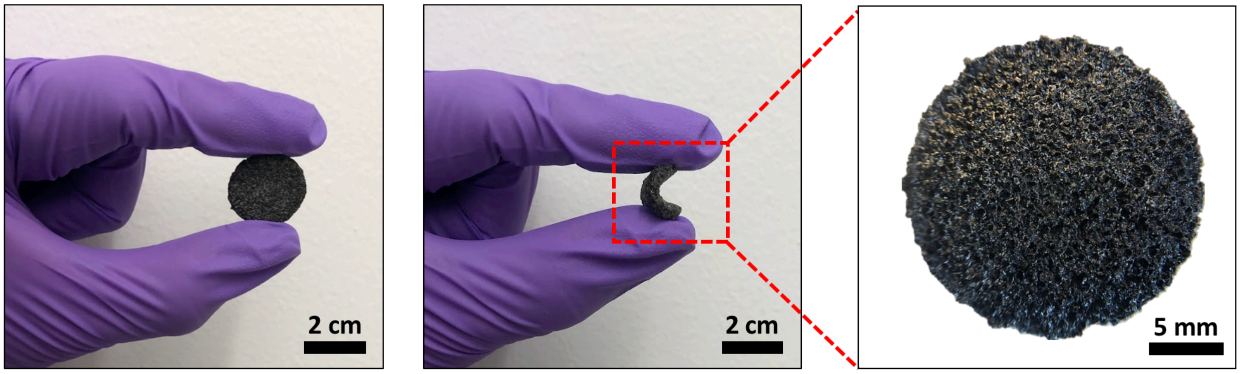

Figure S1. Photographs of the porous PEDOT:PSS/PDMS sponge electrode showing its softness and details of the micropores on the surface.

Placement of the sponge electrodes for electrode-skin contact impedance measurement

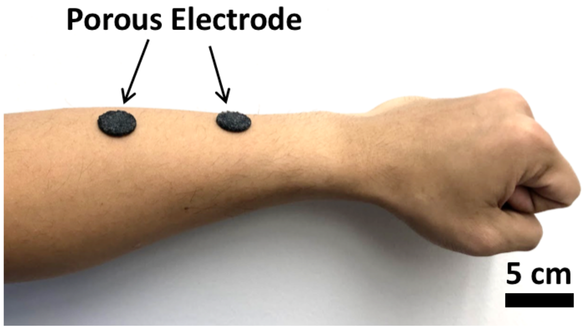

Figure S2. Photograph showing the porous PEDOT:PSS/PDMS electrode placement for electrode-skin contact impedance measurement. Two circular electrodes separated by a distance of 5 cm were placed on the volunteer's arm.

Difference in skin-electrode contact area between the porous electrode and planar electrode when no conductive hydrogel is used

The porous electrode has a typical pore size in the range of 300 – 500 μm and a porosity of 0.6192. When no conductive gel is used, the measured electrode-skin contact impedance is 247 kΩ for the planar electrode, which is slightly lower than the 277 kΩ measured from the porous electrode of the same size. The difference can be attributed to the fact that some micropores in the sponge electrode are only partially in contact with the skin surface, resulting in a slightly smaller skin-electrode contact area compared to the planar electrode.

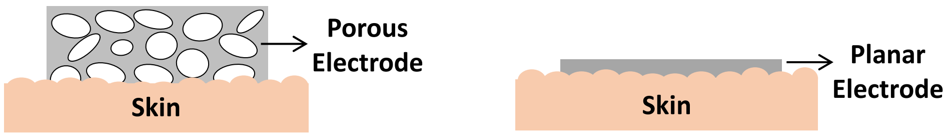

Figure S3. Schematics illustrating the difference in skin-electrode contact area between the porous electrode and planar electrode when no conductive hydrogel is used.

Skin-electrode impedance characterization of the porous electrode under different tensile strain levels

During the experiment, the sponge electrodes were filled with conductive hydrogel and the electrode-skin impedance was measured by placing two electrodes on the skin surface with a separation distance of 5 cm. One electrode was placed on a flat skin surface and the other on the wrist with different bending angles corresponding to different levels of tensile strain from 0% to 20%. The impedance measured at 0% strain was 7.5 kΩ (at a frequency of 10 Hz) and increased only slightly to 8.3 kΩ at 20% strain. The impedance change under tensile strain is relatively small compared to the input impedance of the instrumentation amplifier in our data recording circuit, and thus does not affect the SNR of electrophysiologic signal recording.

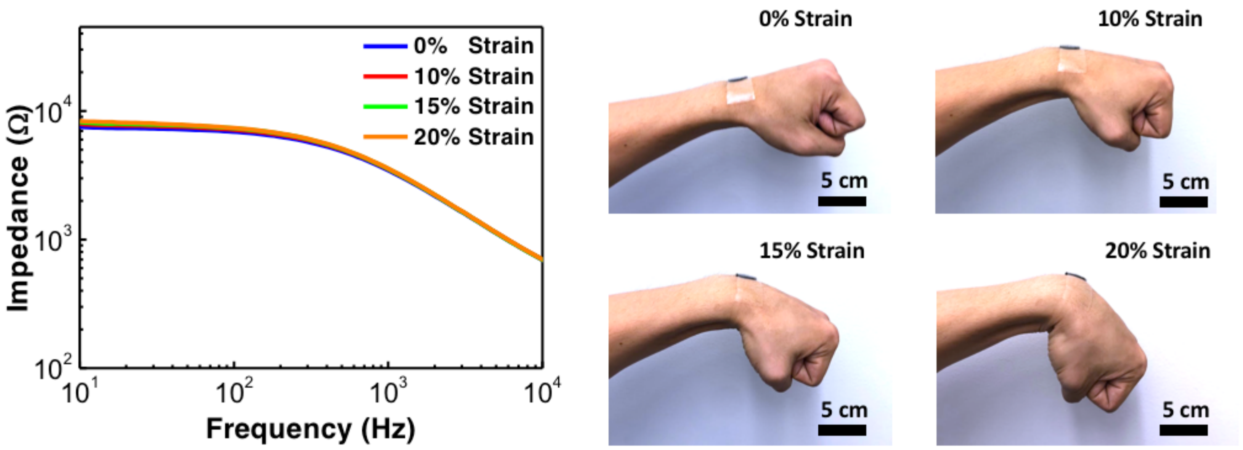

Figure S4. Skin-electrode impedance spectra measured using the porous PEDOT:PSS/PDMS electrodes when the wrist was bent to induce different strain levels in the electrode ranging from 0% to ~20%.

ECG waveforms recorded by the porous electrodes under stretched condition

To test the electrophysiologic signal recording when the porous PEDOT:PSS/PDMS electrodes are under stretched condition, two electrodes were filled with conductive hydrogel and placed on the volunteer's left and right wrists. ECG recordings were conducted under two conditions, one with the wrist lying flat, and one with the wrist flexed to induce approximately a 20% tensile strain in the electrode. Both conditions showed clear ECG signals with periodic QRS complexs and T-peaks, indicating that the sponge electrode can operate under bending conditions.

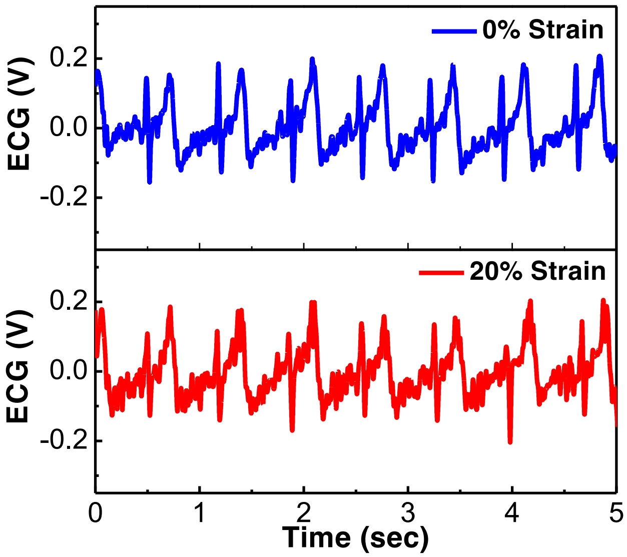

Figure S5. Comparison of ECG signals recorded using the porous PEDOT:PSS/PDMS electrode in flat and stretched conditions.

Porous electrode before and after application of the conductive gel

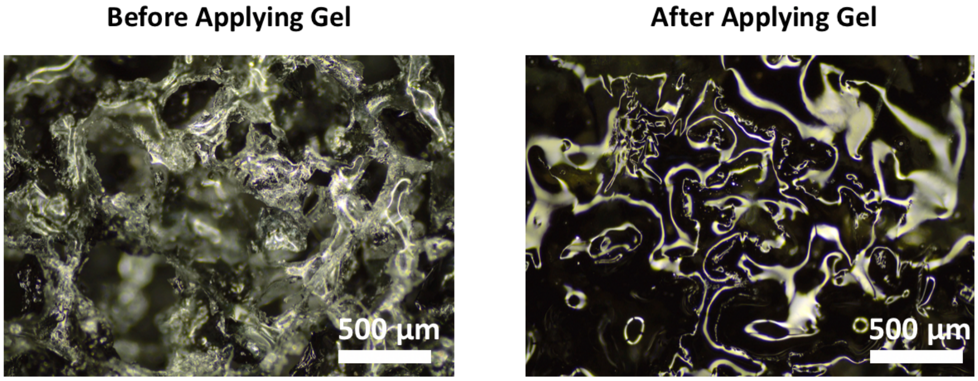

Figure S6. Optical micrographs showing the microstructure of the porous electrode before and after conductive hydrogel application. Once the gel is applied on the electrode, it gets absorbed into the sponge, filling all the micropores inside.

Comparison of gel drying time on porous electrode and planar electrode

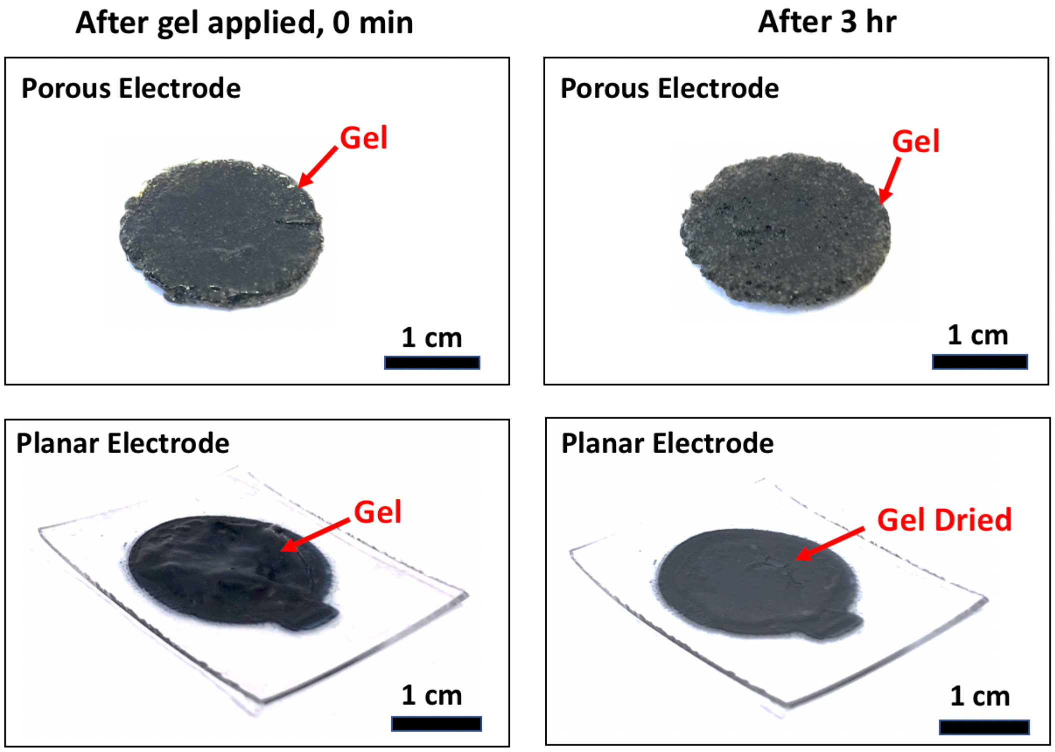

Figure S7. Photographs showing a comparison between the drying times of conductive hydrogel on the porous electrode and planar electrode. Because the gel can be fully absorbed into the micropores inside the sponge electrode, the gel dries much slower compared to the gel that can only be applied on the surface of the planar electrode. After three hours, the gel on the planar electrode surface had completely dried up while the sponge electrode remained wet.

Clinical recording of the electrophysiologic signal from uterine contraction activities using the sponge electrode

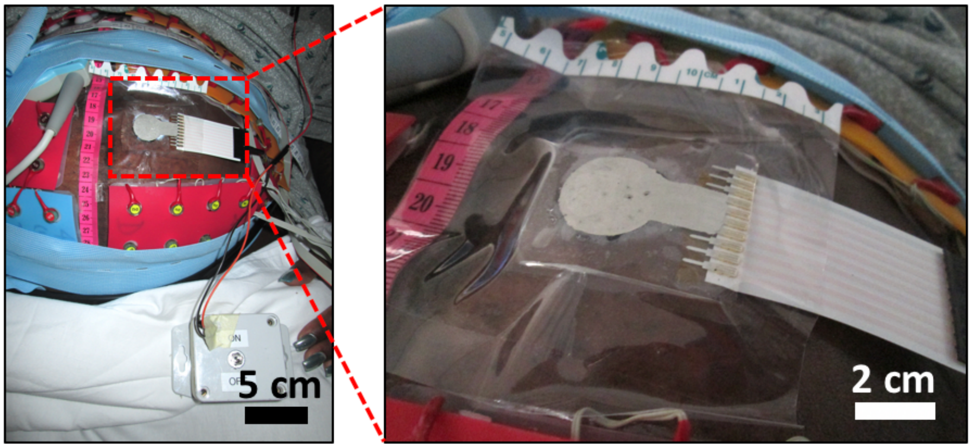

Figure S8. Photographs showing the experiment setup for recording the EMG signal from uterine contraction activities. The picture shows the attachments of the porous electrodes, commercial BioSemi Ag/AgCl electrodes, and TOCO on the anterior abdominal surface of a pregnant woman in labor. A piece of PDMS substrate coated with conductive silver epoxy was applied on the bottom surface of the sponge electrode to form electrical connection between the electrode the data recording unit.

Supplementary Notes

Supplementary Note S1:

Calculation of the internal surface area of all micropores inside a porous electrode

Parameters for porous electrode	
Radius	1 cm
Thickness	2 mm
Porosity	0.6192
Average pore diameter	400 μm

$$Total\ number\ of\ micropores\ inside\ a\ sponge\ electrode$$

$$= \frac{Volume\ of\ an\ electrode\ \times Porosity}{Volume\ of\ a\ single\ micropore}$$

$$= \frac{\pi \times (10^{-2})^2 \times (2 \times 10^{-3}) \times 0.6192}{\frac{4}{3}\pi \times \left(\frac{4\ \times 10^{-4}}{2}\right)^3}$$

$$= 11610$$

$$Total\ surface\ area\ from\ the\ micropores$$

$$=\ Total\ numbers\ of\ micropores\ \times\ Surface\ area\ of\ a\ single\ micropore$$

$$= 11610\ \times\ 4\pi \times (2 \times 10^{-4})^2$$

$$= 5.83\ \times\ 10^{-3}\ (m^2)$$

Supplementary Note S2:

Calculation and comparison of the surface area from a single layer micropores in a porous electrode and the surface area of a conventional planar thin-film electrode

Parameters for porous electrode		Parameter for planar electrode	
Radius	1 cm	Radius	1 cm
Thickness	2 mm		
Average pore diameter	400 μm		

For the sponge electrode, consider only a single layer of micropores filled with conductive gel, calculate the total surface area contributed by these micropores assuming a packing density of 0.8034.[1]

$$\boldsymbol{Total\ surface\ area\ of\ a\ single\ layer\ of\ micropores}$$

$$= Numbers\ of\ pores\ \times\ Packing\ density\ \times\ Surface\ area\ of\ a\ micropore$$

$$= \frac{Cross - sectional\ area\ of\ an\ electrode}{Cross - sectional\ area\ of\ a\ micropore} \times Packing\ density$$

$$\times\ Surface\ area\ of\ a\ micropore$$

$$= \frac{\pi \times (10^{-2})^2}{\pi \times (2 \times 10^{-4})^2} \times 0.8 \times 4\pi \times (2 \times 10^{-4})^2$$

$$= 1.005 \times 10^{-3}\ (m^2)$$

$$\boldsymbol{Total\ surface\ area\ of\ a\ conventional\ planar\ electrode}$$

$$= \pi r^2$$

$$= 3.14 \times 10^{-4}\ (m^2)$$

Supplementary Note S3:

Analysis of the decrease in skin-electrode impedance and increase in surface area offered by the porous electrode compared to the planar electrode.

From Supplementary Notes S1 and S2, the internal surface area of the sponge electrode when considering all the micropores inside the sponge electrode is 0.00583 m^2 and the surface area of the corresponding planar electrode with the same size is 0.000314 m^2. The difference in surface area is 18.56 times. On the other hand, if we consider only the first layer of micropores that are in direct contact with the skin (schematic below), then the surface area is 0.001005 m^2 according to Supplementary Note S2, which is 3.2 times larger than the planar electrode.

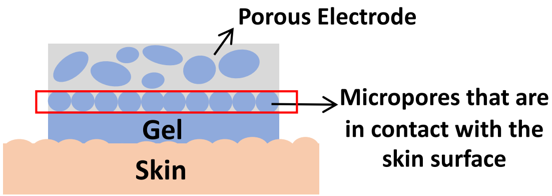

According to Fig. 2h and 2i in the main paper, the electrode-skin contact impedance of the porous and planar electrodes at 10 Hz are 12 kΩ and 63 kΩ, respectively. Thus, the difference in electrode-skin contact impedance between the porous electrode and planar electrode is 5.25 times, which falls between the values of 18.56 times and 3.2 times above. The above results suggest that not all micropores inside the sponge electrode contribute to the electrode-skin impedance, and it is very likely that only the first few layers of micropores that are close to the skin lead to the observed decrease in impedance. The analysis above also explains why the electrode-skin contact impedance does not scale linearly or even change much as the electrode thickness increases from 2 mm to 7.5 mm.

Supplementary References

(1) Melissen, H. Densest Packings of Eleven Congruent Circles in a Circle. *Geom. Dedicata* **1994**, *50*, 15–25.
